# Determinants of translation efficiency in the evolutionarily-divergent protist *Trichomonas vaginalis*

**DOI:** 10.1186/s12860-020-00297-8

**Published:** 2020-07-20

**Authors:** Shuqi E. Wang, Anna E. S. Brooks, Anthony M. Poole, Augusto Simoes-Barbosa

**Affiliations:** 1 School of Biological Sciences, The University of Auckland, Auckland, New Zealand; 2 Department of Microbiology, Immunology, and Molecular Genetics, University of California, Los Angeles, Los Angeles, USA; 3 Maurice Wilkins Centre, The University of Auckland, Auckland, New Zealand; 4 Bioinformatics Institute, The University of Auckland, Auckland, New Zealand

**Keywords:** *Trichomonas vaginalis*, Protozoa, Translation efficiency, Codon usage bias, mRNA secondary structure, Synonymous codons

## Abstract

**Background:**

*Trichomonas vaginalis*, the causative agent of a prevalent urogenital infection in humans, is an evolutionarily divergent protozoan. Protein-coding genes in *T. vaginalis* are largely controlled by two core promoter elements, producing mRNAs with short 5′ UTRs. The specific mechanisms adopted by *T. vaginalis* to fine-tune the translation efficiency (TE) of mRNAs remain largely unknown.

**Results:**

Using both computational and experimental approaches, this study investigated two key factors influencing TE in *T. vaginalis*: codon usage and mRNA secondary structure. Statistical dependence between TE and codon adaptation index (CAI) highlighted the impact of codon usage on mRNA translation in *T. vaginalis*. A genome-wide interrogation revealed that low structural complexity at the 5′ end of mRNA followed closely by a highly structured downstream region correlates with TE variation in this organism. To validate these findings, a synthetic library of 15 synonymous iLOV genes was created, representing five mRNA folding profiles and three codon usage profiles. Fluorescence signals produced by the expression of these synonymous iLOV genes in *T. vaginalis* were consistent with and validated our in silico predictions.

**Conclusions:**

This study demonstrates the role of codon usage bias and mRNA secondary structure in TE of *T. vaginalis* mRNAs, contributing to a better understanding of the factors that influence, and possibly regulate, gene expression in this human pathogen.

## Background

*Trichomonas vaginalis* is a flagellated protozoan that infects the human urogenital tract causing trichomoniasis, the most common non-viral sexually transmitted infection worldwide [1]. Besides its medical importance, *T. vaginalis* is also an organism of interest for studying the early evolution of eukaryotes. Following the revelation of an unexpected repertoire of ~ 60,000 protein-coding genes in the *T. vaginalis* genome [2], a series of transcriptomic and proteomic studies have reported the capability of this parasite to control gene expression in response to different environmental conditions (Reviewed by [3]). These high-throughput data, some of which are publicly available at TrichDB (http://trichdb.org/trichdb/) [4], provide expression evidence for approximately half of the large protein-coding gene repertoire of *T. vaginalis* [5]. In addition, these studies provide useful information about expression abundance at the mRNA and protein levels, allowing further investigations into the molecular mechanisms of translational control in this evolutionarily divergent protist.

Translation efficiency (TE) reflects the rate of protein production per mRNA transcript in a given cellular context [6]. TE is a central parameter in the design of gene sequences for heterologous expression [7] and has been invoked to explain poor correlations between mRNA and protein abundances in both prokaryotes and eukaryotes [8–12]. TE may thus provide means of fine-tuning gene expression, allowing effective investment in the production and recycling of limited cellular resources, such as aminoacyl-transfer RNA (tRNA), ribosome units and ATP [13]. Mechanisms that broadly influence TE have been systematically investigated using synthetic libraries of synonymous reporter genes [7, 14–16] and/or quantitative -omics data of endogenous genes [17–20]. According to these studies, codon usage bias and mRNA secondary structures are the two most common determinants of TE in both prokaryotes and eukaryotes.

Two basic features of molecular systems contribute to codon usage bias. On one hand, codon redundancy (i.e. that 61 codons specify 20 amino acids [21]) potentially allows a wide variety of mRNA sequences carrying different codons to specify the same protein product. On the other hand, the variation on the copy number of tRNA genes [22–24] and the difference in tRNA charging levels [25] lead to an unbalanced supply of isoacceptor aminoacyl-tRNAs in the cytoplasm [26]. As a consequence, the frequent use of codons matching common (i.e. ‘preferred codons’) or rare aminoacyl-tRNA species can lead to different levels of protein production, as delays or pauses in translation are likely to occur at rare codon sites. A strong positive correlation has been observed between the degree of codon bias and the expression level of genes in bacteria and yeasts [27, 28], leading to the development of the Codon Adaptation Index (CAI) [29]. CAI describes the frequency of a gene adopting the ‘preferred’ codons, calculated from a group of highly expressed genes in a genome. For *T. vaginalis*, an informative codon usage table obtained from 189 coding sequences (CDS) is currently available at the Codon Usage Database [30].

In addition to codon usage, secondary structures of mRNAs (i.e. intramolecular base-pairing interactions) also impact TE. RNA secondary structure formation is influenced by the cellular milieu, including the binding of metal ions, proteins and interactions with other RNAs [31]. Complex two- and three-dimensional structural motifs are commonly associated with certain mRNA functional elements or regulatory events, according to recent genome-wide studies (reviewed by [32, 33]). For instance, a significant decrease in structural complexity is generally observed around the boundary between the 5′ untranslated region (UTR) and CDS of mRNAs in most eukaryotes [34–37]. The placement of start codons into unpaired regions potentially allows efficient translation initiation [38]. Similarly, strong regional stability within CDS affects ribosome density and potentially causes ribosome pausing [39]. To date, a range of algorithms have been developed to predict RNA secondary structures based on the primary sequences. They usually scan for all possible structural arrangements of a transcript and identify the one with the minimum free energy (MFE) [40–44]. These MFE calculators are often coupled with a sliding window scheme, allowing the calculation of regional structural stabilities across a series of equal length windows spanning the transcripts [7, 14, 17, 45].

This study systematically investigated the roles of codon usage and secondary structure on the TE of *T. vaginalis* mRNAs. We combined publicly available omics data with in silico analyses to predict how these features impact TE, and subsequently undertook experimental validation of our predictions by examining expression of 15 synonymous iLOV reporters in vivo, which were designed based on our in silico findings. Together, these results reveal key RNA determinants of translational control in this evolutionarily divergent eukaryotic organism.

## Results

### Codon usage bias correlates with TE of mRNAs in *T. vaginalis*

**Fig. 1:**
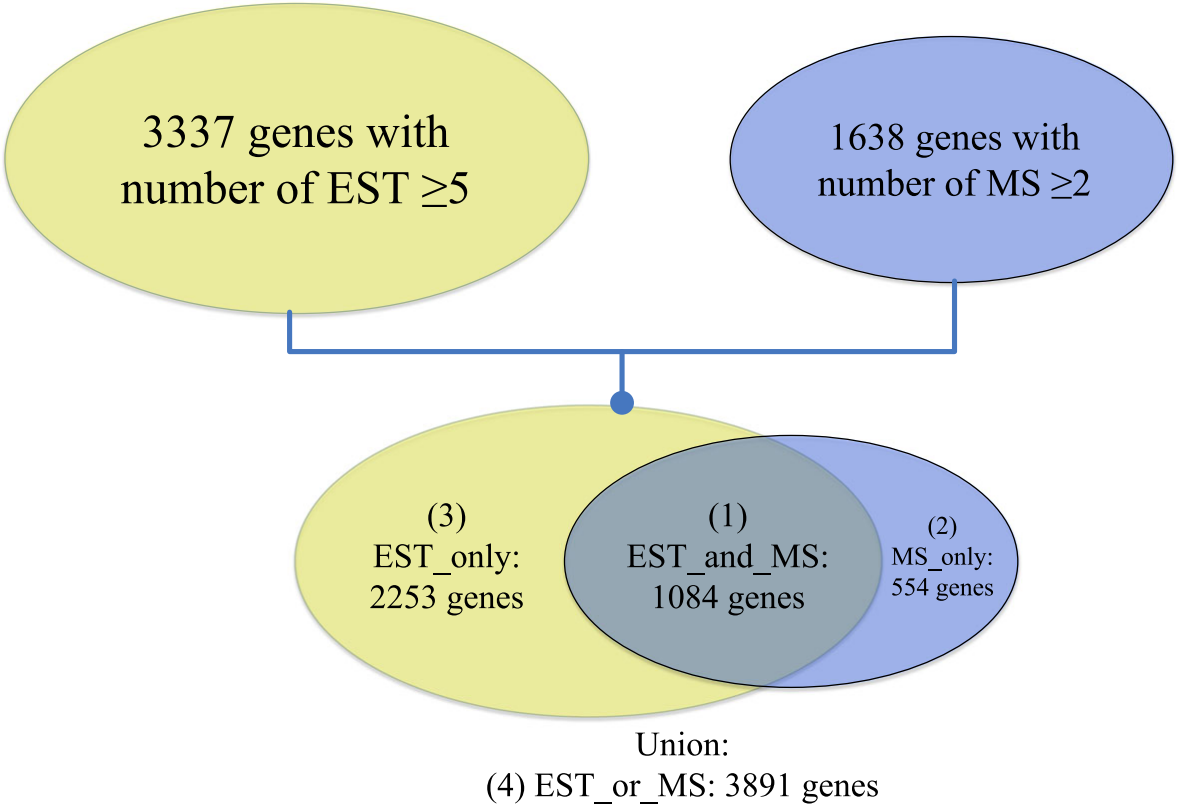
Four datasets of *T. vaginalis* genes with expression evidence were created, based on available transcriptomic (EST) and/or proteomic (MS) data

Sequence and abundance information of *T. vaginalis* protein-coding genes with expression evidence were retrieved from TrichDB [4]. Gene expression at mRNA and protein levels was quantified by ‘number of ESTs’ (nEST) and ‘total number of spectra’ (nMS), respectively. Using the interrogation interface at TrichDB [4], four datasets of *T. vaginalis* genes were collected, based on the different types of expression evidence (Fig. 1). Both types of abundance data (nEST and nMS) are available for genes in Dataset 1 (Fig. 1), allowing us to estimate their TE values, using the following equation: TE = nMS/nEST. By contrast, TE values cannot be calculated for genes in Datasets 2 and 3 because either MS or EST data is missing (Fig. 1). Nevertheless, it was assumed that genes in Dataset 2 (TE approaches +∞) should have higher overall TE than genes in Dataset 3 (TE approaches 0), based on our equation. Dataset 4 is the union of the three datasets, comprising genes with at least one type of expression evidence.

**Fig. 2:**
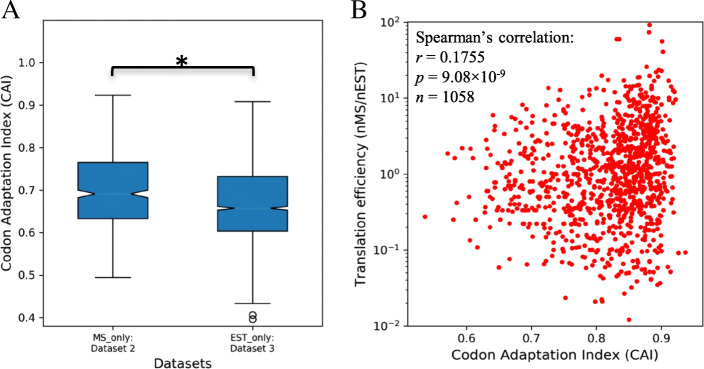
Codon usage bias correlates with TE of protein-coding genes in *T. vaginalis*. Genes in the four datasets (Fig. 1) were subjected to three filters to remove incomplete sequences (Figure S1) and CAI values calculated for each filtered gene. **a** Comparisons of CAI values of genes in Dataset 2 vs Dataset 3. Outliers were plotted as ‘o’ and notches were added to each box, indicating 95% confidence interval of the median. Statistical significance is indicated (*p < 0.01 in an independent t-test). **b** A Spearman’s correlation analysis of TE versus CAI for genes in Dataset 1 was conducted. The *r*, *p* values and sample size n are shown on the top left corner

The open reading frame (ORF) sequences of the genes in the four datasets were isolated and three filters were deployed to remove sequences bearing errors (detailed in Figure S1). CAI values were calculated for error-free gene sequences, using the codon usage table for *T. vaginalis* [30]. The distribution of CAI values of genes in Dataset 2 and 3 was compared, revealing that genes in Dataset 2 have significantly higher CAI values than genes in Dataset 3 (*p* < 0.01, Fig. 2a). The better codon composition of genes in Dataset 2 (i.e. higher CAI) potentially contributes to their higher TE over genes in Dataset 3. This observation may also be explained by the fact that genes with MS data but no EST data must be the result of missing EST data, whereas genes with EST data but not MS data could have expression levels below detection in MS experiments. When the TE values of genes in Dataset 1 were plotted against their CAI values, we observed a weak but significant correlation between these two variables (*r* = 0.1755, *P* = 9.08 × 10^− 9^, *n* = 1058; Fig. 2b) being consistent with other systems since CAI is only one of several factors known to impact on translation [14, 17, 19]. Together, these findings indicate that codon usage bias influences translation of mRNAs in *T. vaginalis*.

### *T. vaginalis* mRNAs exhibit a characteristic structural pattern

**Fig. 3:**
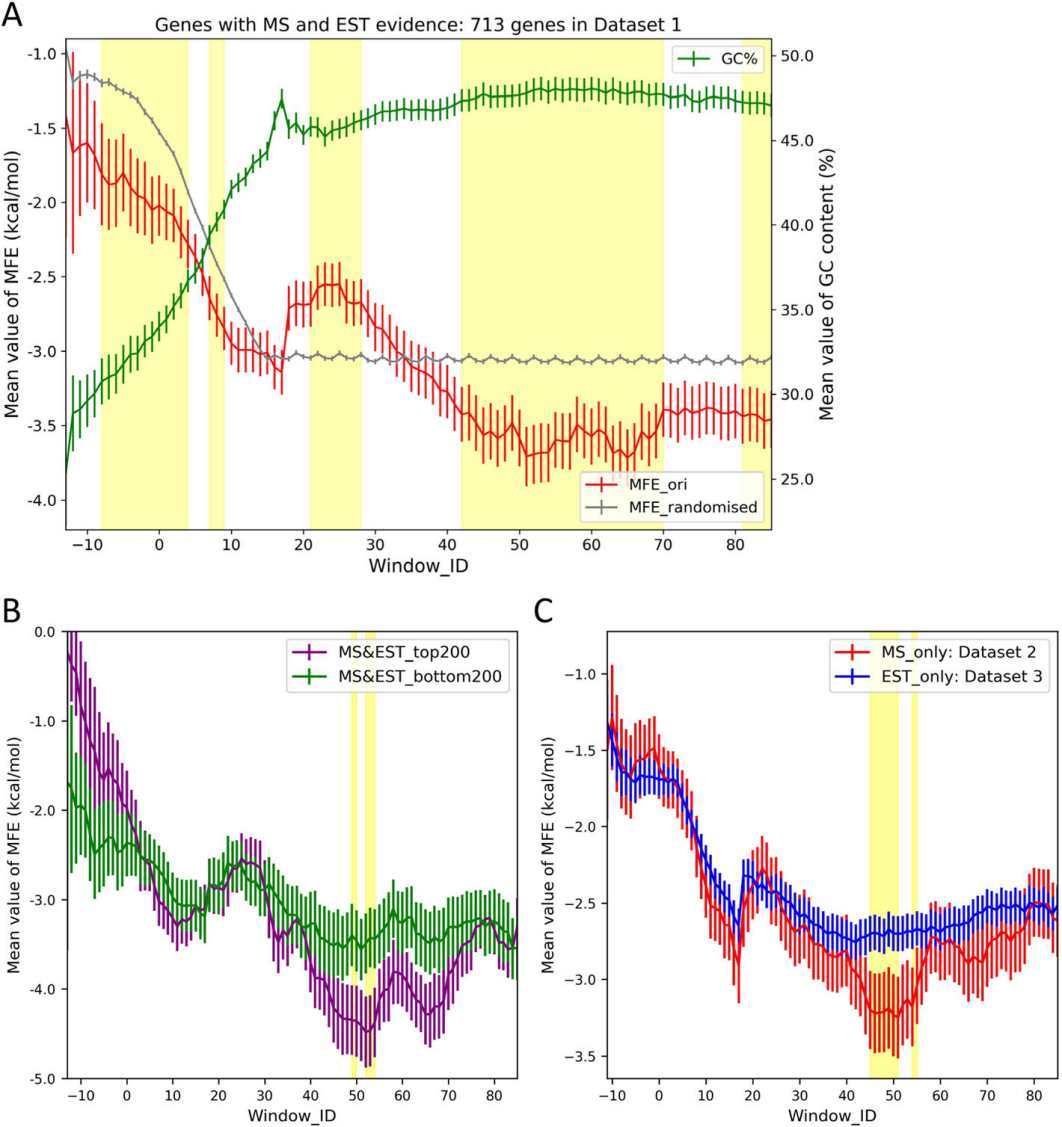
*T. vaginalis* mRNAs exhibit a characteristic structural pattern, which is associated with TE variation. **a** Structural pattern and variation of GC content across the length of *T. vaginalis* mRNAs from Dataset 1. Following the exclusion of genes with unpredictable TSS and combination of genes with > 90% sequence identity (Figure S2), a total of 713 genes were included in this analysis. Genes were sliced into a series of 30 nt wide windows following a sliding window scheme. The index of a window was determined by the position of its central nucleotide relative to the start codon. The value on y axis is the mean MFE value (left) and GC percentage (right) of all sequences from the same window position and the error bar indicates the 95% confidence interval. Values of MFE and GC content for Dataset 1 and the MFE values of the permuted sequences were plotted (red, green and grey curves respectively). Independent t-tests were performed for the two MFE values (original vs. permuted) at the same window position and regions were shaded in yellow if *p* < 0.05, following Bonferroni Correction (α = 0.05). **b**-**c** Comparisons of mRNA folding patterns between *T. vaginalis* genes with different TE: **b** Green and magenta curves represent the top and bottom 200 genes with the highest and lowest TE values in Dataset 1, respectively; **c** Red and blue curves represent genes in Datasets 2 (MS evidence only) and Dataset 3 (EST evidence only), respectively. Independent t-tests were performed for the two MFE values at the same window position and regions were shaded in yellow if *p* < 0.05, following Bonferroni Correction (α = 0.05)

In addition to codon usage bias, a considerable degree of TE variation has been attributed to mRNA secondary structures [14–16]. To examine this potential factor among *T. vaginalis* genes, transcript sequences were first obtained by predicting their transcriptional start site (TSS). This was achieved based on the ubiquitous presence of the core promoter elements Inr and m5, which dictate the TSS in ~ 90% of *T. vaginalis* genes [46]. Genes lacking both elements were removed from each dataset, because their TSS could not be accurately predicted (Figure S2). Sequences with high similarity (> 90% identity) were clustered and represented as the centroid sequence, in order to avoid the over-representation of certain structural features (Figure S2). A high-resolution sliding window scheme was then adopted to slice each transcript into a series of 30 nt windows. Starting from the predicted TSS, these windows moved downstream in 1 nt steps until the 100th nt of the ORF. Each window was indexed based on the location of the central nucleotide relative to the start codon, and MFE was calculated using RNAfold [44]. Genes with at least one type of expression evidence were interrogated by this strategy and MFE values at the same window position were averaged for each dataset. The mRNAs in Dataset 1 possess a reduced structural complexity at the 5′ end, as indicated by high MFE values, followed by a structurally stable region downstream (red line, Fig. 3a). Importantly, the less structured region at the 5′ end includes window position 0, which has the AUG start codon in the centre. This observation agrees with previous reports about the reduced structural stability around the start codon [34–37]. As control, we generated a permuted gene sequence dataset (supplementary text) which we compared to Dataset 1. The curve of permuted genes plotted on the same graph (grey lines, Fig. 3a) exhibited similar or even higher MFE at the 5′ end. Finally, the GC content across the length of the transcripts (green line, Fig. 3a) was found to correlate inversely with MFE values (i.e. high GC correlates with low MFE, red line, Fig. 3a) and these findings were reproducible with genes of Dataset 4 (Figure S3), suggesting that this signal may also be present in those genes with only a single form of expression evidence.

### A characteristic structural pattern is associated with TE of *T. vaginalis* mRNAs

An early study examining intramolecular folding of mRNA in *Escherichia coli* found that MFE in the region − 4 to + 37 nt relative to the start codon correlates strongly with TE of mRNAs [14]. In eukaryotes, a lack of structure surrounding the start codon of mRNAs has also been reported [32]. However, it was found here that neither the first 40 nt of the mRNAs nor the region spanning from − 4 to + 37 nt relative to the start codon contains a local MFE that correlates with TE of *T. vaginalis* mRNAs in Dataset 1 (Figure S4). That said, substantial differences in folding profile are present between *T. vaginalis* genes with high and low TE values (Fig. 3b-c). The top and bottom 200 genes, with the highest and lowest TE values in Dataset 1, showed a substantial degree of MFE divergence at the mRNA 5′ end (Fig. 3b). The top 200 genes possess much higher MFE values than the bottom 200 at the same location (Fig. 3b), indicating the importance of a low structural complexity at the mRNA 5′ end for efficient translation. Additionally, a downstream region (spanning from window 44 to 70) also exhibited divergence of MFE values, where the top 200 genes showed significantly lower MFE than the bottom 200 (*p* < 0.05 following Bonferroni correction, Fig. 3b). Notably, these two discrete regions (i.e. 5′ end and window 44–70) correspond to the 5′ end peak and the two downstream valleys on the general folding pattern curve of *T. vaginalis* mRNAs in Dataset 1 (Fig. 3a). The comparison between the transcripts in Datasets 2 and 3 (Fig. 3c) reiterated this observation, where the valleys were more evident among mRNAs from Dataset 2 (i.e. genes with higher TE than Dataset 3). In conclusion, *T. vaginalis* mRNAs with high TE have a relatively unstructured 5′ end surrounding the start codon AUG and appear to possess stable secondary structures downstream of this.

### Expression of designed synonymous reporter genes in *T. vaginalis* confirms that both codon usage bias and mRNA secondary structure contribute to TE

**Fig. 4:**
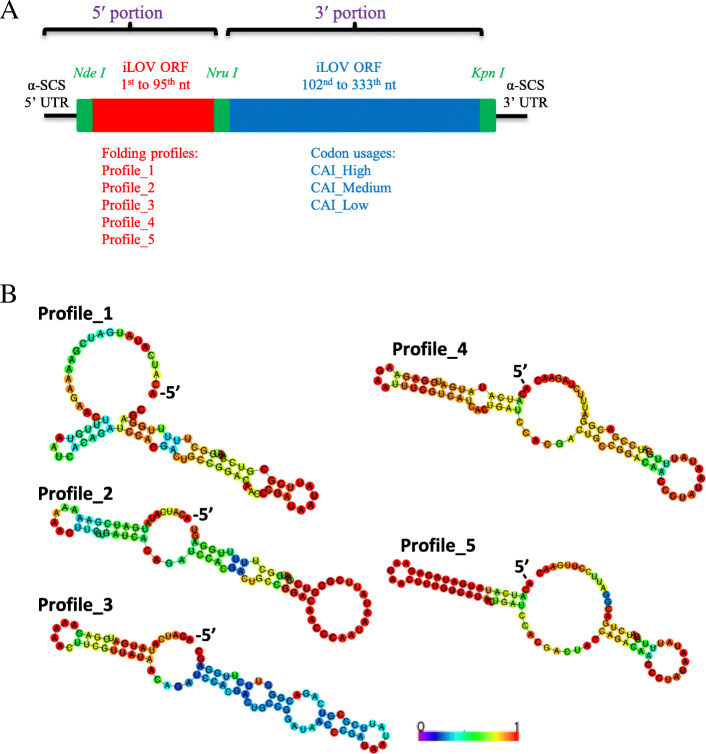
Details of the synthetic iLOV gene library to be expressed in *T. vaginalis.***a** Diagram of the iLOV genes showing 5′ and 3′ portions connected by the *Nru I* site. Five distinct folding profiles and three discrete CAI levels were designed for the sequences at the 5′ and 3′ portions, respectively, without altering the protein product. When combined together after gene synthesis and cloning, these 5′ and 3′ segments produced 15 synonymous iLOV genes. **b** Secondary structures of the five distinct folding profiles were predicted by RNAfold web server (http://rna.tbi.univie.ac.at/cgi-bin/RNAWebSuite/RNAfold.cgi) [42]. The first transcribed nucleotide is pointed on the sequences (− 5′). Color indicates the probability of a particular nucleotide staying in the predicted base-pairing state

In vivo expression of synonymous reporter genes has been utilised to systematically investigate the determinants of TE in other systems [7, 14–16, 19]. Based on the analysis of *T. vaginalis* mRNA features in this study, 15 synonymous iLOV genes were specifically designed to experimentally assess the contributions of codon usage bias and mRNA secondary structure to TE variation in *T. vaginalis*. The iLOV gene, which proved a suitable reporter for *T. vaginalis* under microaerophilic conditions [47], was split into two portions (Fig. 4a). The 5′ portion, composed of the short 5′ UTR derived from the α-SCS promoter [48] and the first 95 nt of the iLOV CDS, was used to create five distinct RNA folding profiles (Fig. 4a-b). To represent structural variations from the one predicted in silico (Fig. 3), sequences of three window positions on the reporter mRNA were carefully chosen and combined (Table 1). Window 8 encompasses the start codon; Windows 51 and 66 enclose the region downstream of the start codon, which is found to be naturally structured among *T. vaginalis* mRNAs (Fig. 3a). The resulting five folding profiles differ in the slope of the MFE curves, ranging from steep downward to steep upward (Table 1). Among them, Profiles 1–3 emulate a gradual change of the natural structural features of *T. vaginalis* mRNA (Fig. 3b), where the 5′ end is less structured than the internal regions. Profiles 4–5, on the other hand, represent folding profiles (i.e. the 5′ end is more structured than the internal regions) that may not exist naturally among *T. vaginalis* mRNAs (Fig. 3b). The 3′ portion, consisting of the last 231 nt of the ORF, was used to create three CAI variants, corresponding to predicted high, medium and low TE (Fig. 4a). Combining the five folding profiles with each of the three CAI variants yielded 15 synonymous iLOV genes (Table 2).

**Table 1:**
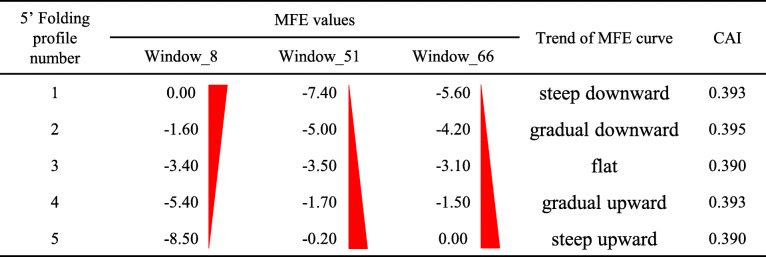
Details of the five folding profiles at the 5′ portion of the iLOV transcript. Each window is indexed by the position of the central nucleotide relative to the start codon. CAI values were calculated based on the first 99 nt of ORF, i.e. the first 33 amino acids of the iLOV protein, and the *T. vaginalis* codon usage table [30]

**Table 2:**
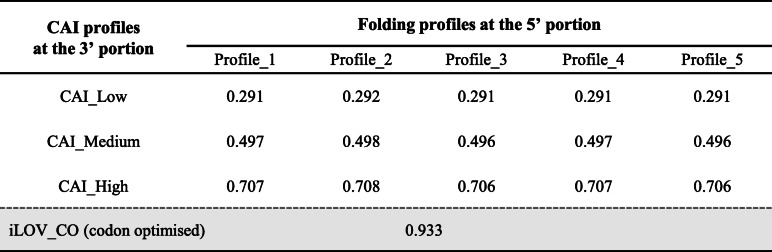
CAI values of the 15 representative iLOV genes, following assembly of their 5′ and 3′ portions, and the codon optimised iLOV_CO [47]

**Fig. 5:**
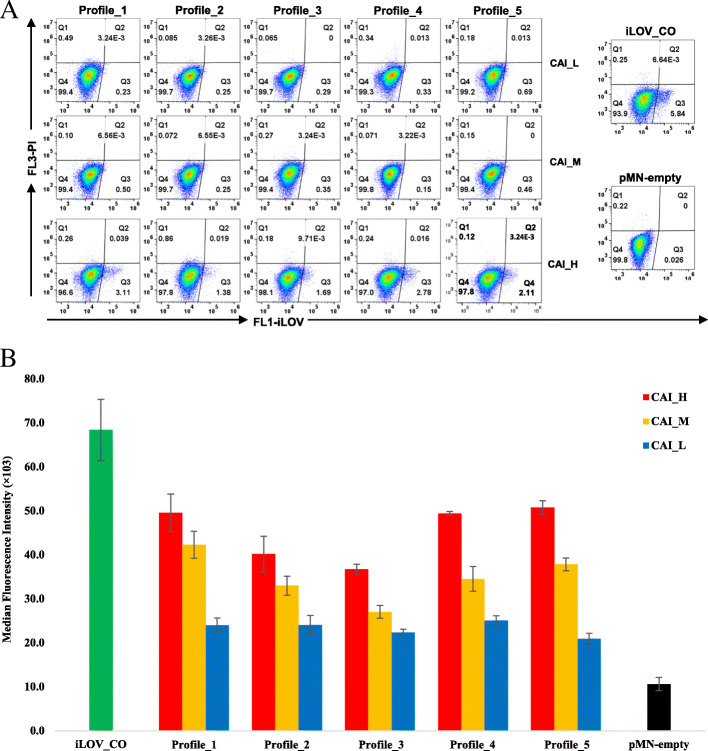
Transient and stable expressions of the synonymous iLOV genes determined by flow cytometry. Data were collected for transient and stable expressions of the 15 synonymous iLOV genes, created by the combinations of five folding profiles (Profile_1 to _5) and three CAI levels (H-high, M-medium and L-low) as per Table 2, from three experimental replicates. pMN-empty and iLOV_CO were used as negative and positive controls, respectively. **a** Transient expression of synonymous iLOV genes in *T. vaginalis*, 8 h post-electroporation. Green fluorescence intensities of transfectants were detected by FL1, and dead cells excluded by FL3 after being stained by 0.2% propidium iodine. Live cells expressing green fluorescence are located in the quadrant Q3 (bottom right). **b** Stable expression of synonymous iLOV genes in *T. vaginalis* after drug selection. One-way ANOVA and Tukey’s range test were conducted to determine the significance of differences between folding profiles and CAI levels (see Additional file: Description of Statistical Analyses)

The 15 synonymous iLOV genes were inserted into the MasterNeo plasmid (pMN) [49] for expression in *T. vaginalis*. iLOV_CO, the codon-optimised iLOV gene (Table 2) [47], was used as a positive control. The pMN-empty vector (i.e. without an integrated reporter gene), was used as a negative control. *T. vaginalis* transfectants for all 17 plasmids were examined by flow cytometry for transient and stable expression of the iLOV genes. At 8 h post-electroporation, green fluorescent signals were only detected in samples expressing iLOV with high CAI levels, including the positive control iLOV_CO (Fig. 5a). Among these, no obvious variations in expression level were observed across the five folding profiles (Fig. 5a). iLOV genes with low or medium CAI levels produced no detectable green fluorescence during the course of transient expression, regardless of the folding profile. We next quantified the transient expression of fluorescence by multiplying the percentage of dots located in quadrant Q3 and the median value of this population on the FL1 axis (see Fig. 5a). The results from three independent transfection assays indicated that iLOV genes with high CAI level appear to produce a much stronger transient expression of green fluorescence compared to their counterparts with the same folding profile (Figure S5), although these differences lack statistical significance following a Tukey’s range test (*p* > 0.05, yellow shaded cells in Table S1).

Following drug selection, stable transfectants were sampled for flow cytometry analysis. The average fluorescence intensity, obtained from three independent transfection assays, was determined for each plasmid (Fig. 5b). All 15 synonymous iLOV genes produced detectable green signals above background (i.e. pMN-empty transfectants) but lower than iLOV_CO (Fig. 5b). These differences are statistically significant (*p* < 0.05, grey shaded cells in Table S1). Importantly, when synonymous genes of the same folding profile were compared, the fluorescence intensity decreased from high through low CAI, without exception (Fig. 5b). The majority of these differences (12 out of 15 cases) are statistically significant (*p* < 0.05, yellow shaded cells in Table S2) following correction for multiple testing (see Additional file: Description of Statistical Analyses). Overall, these findings indicated that codon usage significantly affects TE in *T. vaginalis*.

In addition to CAI, the mRNA secondary structure folding profiles appear to impact TE of *T. vaginalis* mRNAs (Fig. 5b). Comparison of profiles 1 through 3 indicated a gradual decrease in the reporter fluorescence intensities (Fig. 5b). This was particularly evident for cells expressing iLOV genes with high and medium CAI (4 of the 6 p < 0.05, green shaded cells in Table S2). The transition from profiles 3 through 5, on the other hand, led to an increase in fluorescence intensities (Fig. 5b). This was again evident for cells expressing iLOV genes with high and medium CAI. Profiles 4 and 5 deviate from the MFE profiles of *T. vaginalis* mRNAs in our dataset (Fig. 3), but had a similar effect on fluorescence intensity to profile 1 (which most closely resembles the MFE profiles from natural mRNAs in Fig. 3). Collectively, these findings indicated that the mRNA secondary structure also influences TE in *T. vaginalis*.

## Discussion

Translation efficiency (TE) implies that the mRNA itself contains information that directly affects the protein synthesis rate during translation. To investigate factors that potentially modulate TE in a particular organism, previous studies have relied on in silico analyses of expression abundances of endogenous genes [17–20] and/or in vivo expression of synonymous reporters [7, 14–16]. According to Tuller et al. [17], these two strategies interrogate TE at global and local levels respectively, considering the overall sample size of the reference genes (entire genome versus one single reporter). While some factors may modify TE in a uniform manner across the entire transcriptome regardless of the expression levels of individual genes, others can only act upon a particular set of transcripts [17]. Our study combined computational and experimental methods, in order to assess the contributions of codon usage bias and mRNA secondary structure to TE variation in the evolutionarily-divergent eukaryote *T. vaginalis*.

To evaluate the contribution of codon usage bias to the TE of endogenous genes, we focused on a core set of 1058 *T. vaginalis* genes with solid transcriptomic (nEST ≥5) and proteomic (nMS ≥ 2) expression evidence (Dataset 1, Fig. 1). A significant correlation was observed between TE (nMS/nEST) and CAI of these genes (Fig. 2b), indicating that codon usage bias tunes TE level for *T. vaginalis* protein-coding genes globally. Additional comparisons were made on the distributions of CAI values of endogenous genes supported by either type of expression evidence. Genes in Dataset 2, with strong MS but little or no EST evidence, were found to possess significantly higher CAI values than the opposite group in Dataset 3 (Fig. 2a), corroborating the impact of codon usage bias on TE in *T. vaginalis*.

To examine a possible role for mRNA secondary structure, we took advantage of the fact that the TSS of genes in this protist can be predicted with confidence based on the ubiquitous presence of conserved core promoter elements that dictate transcription initiation [46]. We first interrogated mRNA transcripts in Datasets 1 and 4 for global folding patterns. We observed that *T. vaginalis* mRNAs possess weak structuring at their 5′ ends (red curves, Fig. 3a), irrespective of the individual abundances. This structural feature, observed in most eukaryotic cells investigated to date [34–37], could potentially enable rapid translation initiation of the mRNA transcripts. The short 5′ UTR of *T. vaginalis* mRNAs and the AUG start codon were found to fall into a region with low GC content (green curves, Fig. 3a). Our results suggest that natural selection has shaped *T. vaginalis* genes to have a low level of structural complexity around the start codon by depressing GC content specifically at the 5′ ends of their mRNAs. Our data do not allow us to establish exactly how this profile relates to TE, but one possibility is that this profile makes the 5′ end accessible for capping or aids the efficient assembly of the translation initiation complex.

In contrast to observations in *E. coli* [14], we did not detect a direct correlation between MFE around the start codon and the TE of *T. vaginalis* mRNAs (Figure S4). Instead, our categorisation of genes in Dataset 1 based on expression abundances, revealed that the top and bottom 200 genes exhibited apparent MFE divergences at the 5′ end as well as at a downstream region (from window position 44 to 70; Fig. 3b, yellow shaded). Genes with higher TE exhibited a less structured 5′ end and, conversely, a more structured downstream region than genes with lower TE. This finding was also observed when comparing Datasets 2 and 3 (Fig. 3c). These observations suggest that the structural stability at two discrete positions, rather than the 5′ end alone, shapes the TE of *T. vaginalis* mRNAs. Our data thus allow us to conclude that a characteristic structural pattern, defined by a poorly-structured 5′ end closely followed by a more stably-structured downstream region, facilitates efficient translation of *T. vaginalis* mRNAs. The minimal structural complexity at the 5′ end is likely to allow rapid ribosome binding and assembly, while the stably-structured downstream region may potentially act as an insulator preventing interactions of the unpaired 5′ end nucleotides with downstream regions of the mRNA.

To validate our computational characterisation experimentally, we designed a collection of synonymous iLOV genes that were expressed in *T. vaginalis*. Unlike the in silico investigations, this local scale survey used in vivo protein expression levels to determine TE. Importantly, the synonymous reporter genes were transcribed under the same regulatory context allowing control of the mRNA level. The 15 synonymous iLOV genes, along with the codon-optimised variant (iLOV_CO) [47], represented four expected tiers of TE, based on manipulation of the CAI (Table 2). As predicted, use of ‘preferred codons’ in the five iLOV mRNAs with high CAI plus the iLOV_CO led to a faster production and accumulation of the iLOV protein, to a level that was detectable by flow cytometry (Fig. 5a). By contrast, little or no fluorescent signals were produced during the course of transient expression by the remaining 10 transcripts that rely on rare aminoacyl-tRNAs for translation (Fig. 5a). During stable expression, the CAI directly impacted the fluorescence intensities, as was clear from comparison of synonymous iLOV genes with the same folding profile (Fig. 5b). Together, these findings indicate that codon usage bias has a major role in determining the TE of *T. vaginalis* mRNAs.

The regulatory effects of mRNA secondary structures were also interrogated using our synthetic library of iLOV genes. Based on the predicted structural features of *T. vaginalis* mRNAs (Fig. 3), three discrete regions of the transcripts (represented by Window_8, Window_51 and Window_66; Fig. 3a) were altered to create five distinct folding profiles (Table 1). From profiles 1 through 5, the MFE values were gradually dropped at Window_8, and simultaneously increased at the two downstream windows (Table 1 and Fig. 4). The structural changes from profiles 1 to 3 (Fig. 4) emulated the transition of *T. vaginalis* mRNAs from high to low TE (Fig. 3b). These progressive changes generated an inversion of the MFE profiles (Table 1), with profiles 4 and 5 exhibiting MFE values at the 5′ ends lower than at the downstream region. Endogenous *T. vaginalis* mRNAs do not seem to carry this inverted shape of the MFE curve 5′ to 3′, i.e. from down to upward (Fig. 3). Notably, the effect of codon usage bias could be separated from structural effects on TE. Structure was unable to counteract the impact from codon usage at the transient expression stage (Fig. 5a), but the effect of secondary structures on TE became evident during stable expression of the reporter genes (Fig. 5b). Interestingly, this was not the case for low CAI iLOV genes, which showed no significant change in fluorescence intensity across the five secondary structure profiles (Fig. 5b and Table S2). This suggests that, while secondary structure can augment TE for genes with CAI profiles that result in medium or high expression, it has little or no impact on genes whose CAI profiles result in poor expression. Given that translation initiation is known to be rate-limiting [50], the accessibility of the 5′ end might help increase TE. However, for mRNAs with low CAI, we suspect that translation elongation is also affected to an extent that mRNA structural changes have no additional or detectable impact on TE.

Translation is central to gene expression and an important checkpoint for protein production in a cell. While codon usage bias and mRNA secondary structures have been claimed to strongly influence TE [14–17], post-transcriptional mechanisms of gene control might also be involved. Processes that control polyadenylation and the ‘close loop’ configuration of mRNAs, mediated by proteins that bind to responsive elements found in the UTRs, may affect mRNA stability and thus translation [51, 52]. Moreover, sequence elements upstream of a gene of interest, such as introns and ORFs, have been shown to influence TE [53–56]. Thus, it may well be that other factors have a significant role in determining TE in *T. vaginalis*. However, it is worth noting that there are few examples of post-transcriptional control in *T. vaginalis* [57, 58], while UTRs are remarkably short and introns are rare [5, 46]. Together with the widespread distribution and utilization of conserved core promoter elements [46], these genetic features make *T. vaginalis* an interesting model for the study of TE in eukaryotes.

## Conclusions

Using complementary in silico and in vivo approaches, this study systematically investigated the impact of codon usage bias and mRNA secondary structure on TE of *T. vaginalis* mRNAs. In addition to guiding optimal gene expression in this parasite, the results here provide new information that leads to a more comprehensive understanding of the mechanisms by which gene expression is controlled in *T. vaginalis*. Despite having a translation machinery that is rather typical of eukaryotes, *T. vaginalis* and other evolutionarily divergent protozoans such as *Giardia* [59] produce mRNAs with short 5′ UTRs. This study indicates that *T. vaginalis* mRNAs contain information at the level of both sequence and structure that impact their expression, adding a novel layer to our understanding of gene regulation in this divergent unicellular eukaryote.

## Methods

### Data retrieval, clean-up and TE calculation

Expression abundances of *T. vaginalis* protein-coding genes at mRNA and protein levels, quantified by nEST and nMS respectively, were downloaded from TrichDB [4]. A threshold was set for the minimum nEST or nMS of each gene, resulting in 3337 genes with nEST ≥5 and 1638 genes with nMS ≥ 2 (Fig. 1). Using the panel ‘My Strategies’ at TrichDB, four datasets of *T. vaginalis* genes with differential expression evidence were isolated (Fig. 1). Dataset 1 and Dataset 4 consist of 1084 and 3891 *T. vaginalis* genes respectively, which represent the intersection and union of the two above groups (Fig. 1). Dataset 2 and Dataset 3 comprise 554 and 2253 genes, respectively, which were supported by either type of expression evidence (Fig. 1). Gene sequences in each dataset were downloaded from TrichDB in FASTA format. A sequence was removed from the datasets if it (i) does not have a start codon and/or stop codon at the expected position, (ii) possesses ambiguous nucleotides (bases other than ‘ATCG’), or (iii) has an internal stop codon within the ORF (Figure S1). Number of genes in each dataset after clean-up is shown in Figure S1. TE values were calculated for genes in Dataset 1 as the ratio of nMS/nEST (Figure S1).

### Correlation analysis between codon usage bias and TE of *T. vaginalis* mRNAs

The ORF sequence of each gene was isolated and subjected to CAI calculation using the local version of CAIcal SERVER [60], based on *T. vaginalis* codon usage table downloaded from Codon Usage Database [30] (Figure S1). Boxplots were drawn to show the distributions of CAI values of Dataset 2 and 3 separately. A pairwise comparison was made for CAI values in Dataset 2 and 3, using an independent t-test. TE value of each gene in Dataset 1 was plotted against its CAI and a Spearman’s Correlation Analysis was conducted to determine the statistical dependence between these two parameters (see Additional file: Description of Statistical Analyses).

### General folding profile of *T. vaginalis* mRNAs

A filter was created to scan the 20 nt upstream region in the 5′ UTR and return genes carrying the signature sequence ‘CCTTT’ of the m5 core promoter element [46] (Figure S2). Similarly, another filter was used to scan the remaining sequences and return genes with the Inr core promoter element (‘HCAHW’ [48, 61]) in the 30 nt upstream region (Figure S2). The remaining minorities transcribed by neither elements were discarded, as their specific TSS could not be predicted. Genes transcribed by either m5 or Inr were trimmed from the putative TSS, as underlined here (‘HCAHW’ for inr; ‘CCTTT’ for m5), to the 100th nt of the ORF (Figure S2). To avoid over-representation of certain folding patterns due to the duplicated genes in *T. vaginalis* genome [2], uclust_fast algorithm [62] was employed to cluster sequences in each dataset with more than 90% identity and produce a centroid representative for each cluster (Figure S2). A sliding window scheme [17, 45] was used to slice the putative transcripts in Dataset 1 and Dataset 4 into a series of 30 nt windows, via continuous frame shift with steps of 1 nt (Figure S2). Each window was indexed by the position of its central nucleotide relative to the start codon. RNAfold program in ViennaRNA Package 2.0 [44] was used to calculate the MFE value of each window (Figure S2). Moreover, each transcript was permuted for 100 times using a strategy modified from early studies [17, 45] (Figure S2). Briefly, the 5′ UTR and ORF of a gene were subjected to mononucleotide and triplet codon shuffling, respectively. The permuted sequences were also interrogated by the same sliding window scheme and MFE calculation. The mean MFE values of the original and permuted sequences across the transcript positions were plotted on the same graph, with error bars added showing 95% confidence interval. A series of t-tests were carried out between MFE values of the original and permuted sequences at the same window position. Reported *p* values were adjusted by Bonferroni Correction (see Additional file: Description of Statistical Analyses). At last, the average GC content at each window position was also calculated and plotted on the same graph.

### Relationship between the RNA folding profile and TE of *T. vaginalis* mRNAs

The first 40 nt, or the region spanning from − 4 to + 37 nt relative to the start codon was isolated from each putative transcript in Dataset 1 and subjected to MFE calculation. The two MFE values of each transcript were separately plotted against its TE and a Spearman’s Correlation Analysis was conducted to determine the statistical dependence between each of the MFE values and TE. Genes from Dataset 1 were ranked based on their TE values and 200 genes with the highest (Top 200) or the lowest (Bottom 200) TE were isolated and subjected to the same sliding window scheme and MFE calculation (Figure S2), as per above. Folding profiles of these two groups of genes were plotted on the same graph for comparison. Likewise, folding profiles of genes in Dataset 2 and Dataset 3 were also compared. A series of t-tests were carried out between the two groups of MFE values at the same window positions and the resulting *p* values were adjusted by Bonferroni Correction (see Additional file: Description of Statistical Analyses).

### Python scripts

Python programming language was widely used in this study to process gene sequences, make calculations, perform statistical analysis and plot data on graphs. All Python scripts in this study have been uploaded to https://github.com/Huaqiedward.

### Selection and assembly of representative iLOV genes for the synthetic library

Amino acid sequence of iLOV protein was submitted to Backtranambig tool in EMBOSS [63] for a back-translation and a DNA sequence full of ambiguous codes was generated, representing all possible synonymous genes. The position 96–101 of this back-translated ORF was fixed as ‘TCGCGA’, the restriction site of *Nru I* endonuclease, without altering the amino acids specified (Fig. 4a). The regions upstream (5′ portion) and downstream (3′ portion) of the *Nru I* site were used to create RNA folding and CAI variations respectively. The 5′ UTR derived from the promoter of α-succinyl CoA synthetase (α-SCS) gene in *T. vaginalis* (7 nt in length) was linked to the 5′ end and the joined sequence was sliced by the same sliding window scheme as described above. Window 8, 51 and 66, containing ambiguous DNA codes, were expanded into all possible unambiguous sequences. MFE values of these unambiguous sequences were calculated and the distribution of MFE values was analysed for each window position separately. Five representative unambiguous sequences were chosen for each window position, representing five discrete MFE levels (Table 1). These selected sequences were combined together in a way as shown in Table 1, resulting in five distinct folding profiles. Moreover, nucleotides outside Window 8, 51 and 66 were also carefully chosen, in order to balance the overall CAI values of these 5′ portions (Table 1). Each of these 5′ portions was submitted to RNAfold web server [42] to generate a secondary structure illustration. For the 3′ portion, synonymous sequences were designed using OPTIMIZER web server [64], to produce three ORF segments with high, medium and low CAI levels. As a final assembly, the 5′ portions were joined with each 3′ portion, which resulted in 15 synonymous iLOV genes representing five structural variants and three codon usage variants (Table 2).

### Gene synthesis and plasmid construction

The 15 synonymous iLOV genes were chemically synthesised by Integrated DNA Technologies (IDT) and inserted to pMN [49] for expression under the regulation of *T. vaginalis* α-SCS UTRs via *Nde I* and *Kpn I* sites. The sequence of each synonymous iLOV gene was confirmed by Sanger sequencing. Additionally, another pMN expressing the codon-optimised iLOV gene (iLOV_CO) [47], and pMN-empty were included as controls.

### Transient and stable expressions of the synonymous iLOV genes

A standard electroporation protocol [49] was used to introduce each plasmid to *T. vaginalis* G3 strain cells. All 17 plasmids were examined in three independent transfection assays. Briefly, wild type G3 strain *T. vaginalis* cells were cultured in complete Diamond’s medium at 37 °C, until reaching a concentration of 1–2 × 10^6^ cells/ml. Cells were harvested by centrifugation at 3000 g for 20 min at 4 °C and resuspended in cold fresh Diamond’s medium to a concentration of 8.33 × 10^8^ cells/ml. Aliquots of 300 μl cell suspension were transferred into 0.4 cm electrocuvettes (Bio-Rad), mixed with 60 μg plasmid and electroporated at 350 V with 975 μF capacitance using a Bio-Rad Gene Pulser II. Electroporated cells were transferred to 50 ml pre-warmed Diamond’s medium, incubated at 37 °C. At 8 h post-electroporation, cells were sampled from the supernatant, pelleted by centrifugation, resuspended in phosphate-buffered saline (PBS) and taken for analysis of transient expression. After sampling of cells for transient expression analysis, G418 was immediately added to cell culture at a concentration of 200 μg/ml to perform drug selection. After 18–24 h, the pellets containing dead cells and debris in each tube were removed. Cells in the supernatant were then recovered by centrifugation and resuspended in fresh complete media containing G418. Once cells grew to a concentration of 0.5–1 × 10^6^ cells/ml in the presence of G418 (normally 4–6 days post-transfection), they were passaged daily always in the presence of G418. After being passaged for 1 week, cells were harvested by centrifugation, resuspended in PBS and taken for analysis of stable expression.

### Flow cytometry analyses

Both transient and stable expression analyses of the iLOV genes were carried out with an Accuri C6 Flow Cytometer, BD Biosciences. Green fluorescence intensity was detected by FL1, and dead cells excluded by FL3 after being stained by 0.2% propidium iodine (PI). A protocol was set up to count a total of approximately 30,000 live cells per sample. At the transient expression stage, the presence of live and FP-positive cells was confirmed by dots (individual cells) located in quadrant Q3. The strength of fluorescent signals released from each sample during transient expression was quantified by multiplying the percentage of dots located in quadrant Q3 and the median FL1 value of all dots in Q3. At the stable expression stage, the fluorescence intensity of every single cell was measured by FL1 and the median fluorescence intensity value of the entire cell community in each transfected sample was calculated. For both transient and stable expression data, One-way analysis of variance (ANOVA) and Post hoc analysis (Tukey method) were performed (see Additional file: Description of Statistical Analyses) to determine the significance of difference between different folding profiles and CAI levels or between each of the 15 synonymous iLOV genes and controls.

## Supplementary information

**Additional file 1: Figure S1.** Workflow for the investigation of codon usage bias as a TE determinant in *T. vaginalis.***Figure S2.** Workflow for the investigation of mRNA secondary structure as a TE determinant in *T. vaginalis.***Figure S3.** Structural pattern and variation of GC content across the length of *T. vaginalis* mRNAs from Dataset 4. **Figure S4.** Spearman’s correlation analyses did not reveal a direct association between TE and MFE at 5′ end or surrounding the AUG start codon of *T. vaginalis* mRNAs from Dataset 1. **Figure S5.** Transient expression of the synonymous iLOV genes from three independent transfections. **Table S1.** Pairwise comparisons across transient expression illustrated on Fig. S5. **Table S2.** Pairwise comparisons acrosss stable expression illustrated on Fig. 5b.

## Data Availability

The datasets used and/or analysed during the current study are available from the corresponding author on reasonable request.

## Abbreviations

UTR: Untranslated Region
TE: Translation Efficiency
CAI: Codon Adaptation Index
iLOV: The fluorescent flavoprotein Improved LOV derived from Light, Oxygen, or Voltage domains
CDS: Coding Sequences
MFE: Minimal Free Energy
EST: Expressed Sequence Tag
MS: Mass Spectra
ORF: Open Reading Frame
TSS: Transcription Start Site
pMN: MasterNeo plasmid
