# Supplementary material for: Determinants of translation efficiency in the evolutionarily-divergent protist Trichomonas vaginalis
Source: BMC Mol Cell Biol. 2020 Jul 20;21:54. doi: 10.1186/s12860-020-00297-8 (PMC7370421; doi:10.1186/s12860-020-00297-8)
Supplement: Supplementary file 1 — Additional file 1: Figure S1. Workflow for the investigation of codon usage bias as a TE determinant in T. vaginalis.Figure S2. Workflow for the investigation of mRNA secondary structure as a TE determinant in T. vaginalis.Figure S3. Structural pattern and variation of GC content across the length of T. vaginalis mRNAs from Dataset 4. Figure S4. Spearman’s correlation analyses did not reveal a direct association between TE and MFE at 5′ end or surrounding the AUG start codon of T. vaginalis mRNAs from Dataset 1. Figure S5. Transient expression of the synonymous iLOV genes from three independent transfections. Table S1. Pairwise comparisons across transient expression illustrated on Fig. S5. Table S2. Pairwise comparisons acrosss stable expression illustrated on Fig. 5b. [file 12860_2020_297_MOESM1_ESM.pdf]

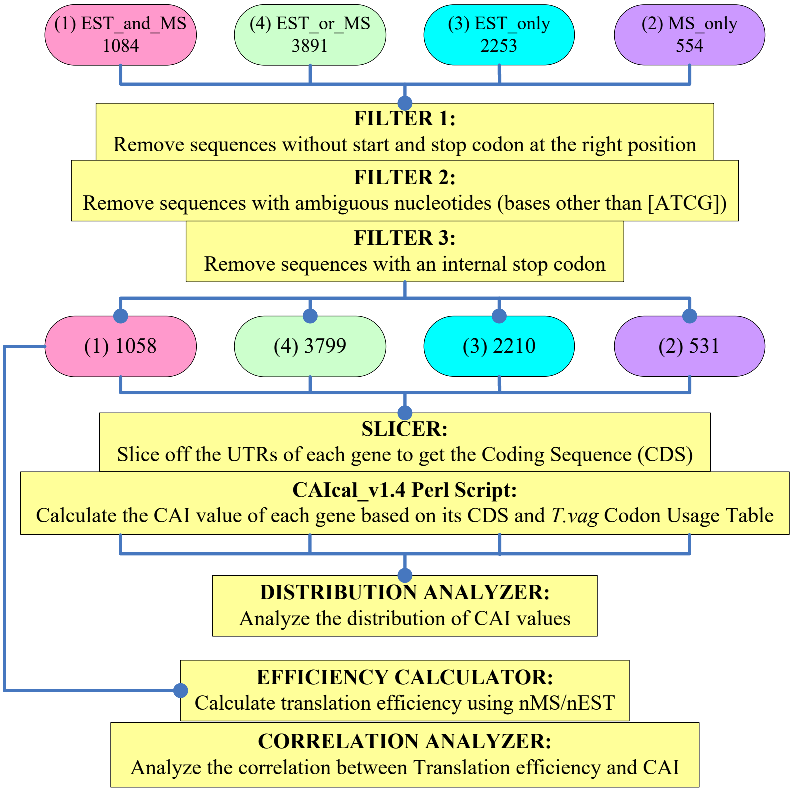

Fig. S1 Workflow for the investigation of codon usage bias as a TE determinant in *T. vaginalis*. Three filters were set to remove incomplete sequences from the four datasets (as per Fig. 1), labelled here (1) to (4) and color-coded for convenience. The numbers of genes in each dataset before and after data clean-up are shown. The CAI value of each gene was calculated based on its ORF sequence and the *T. vaginalis* codon usage table [30]. The distributions of CAI values of the four datasets were analysed. For genes in Dataset 1, TE values were quantified as a ratio of nMS/nEST. The statistical correlation between TE and CAI was determined by a Spearman's Correlation Analysis using genes in Dataset 1.

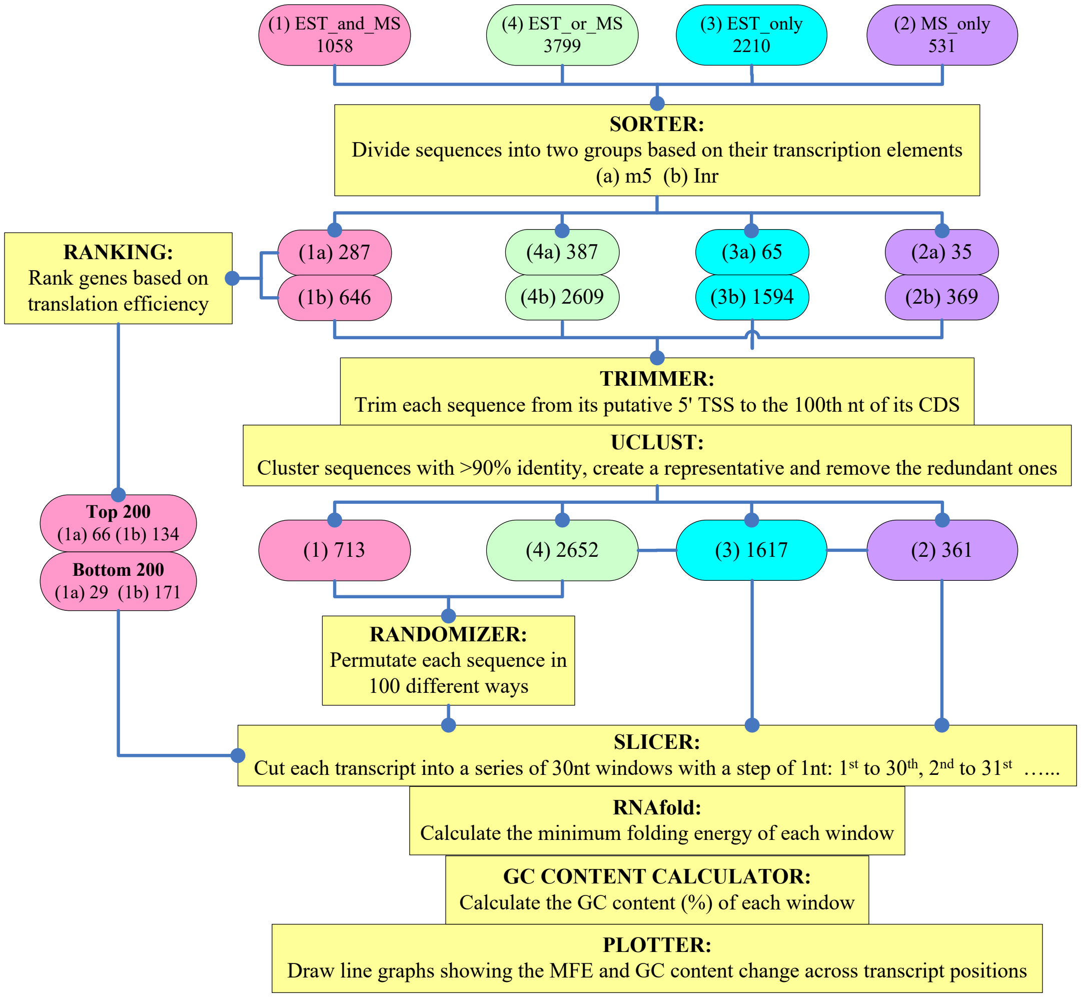

Fig. S2 Workflow for the investigation of mRNA secondary structure as a TE determinant in *T. vaginalis*. The 5′ UTR of genes in the four datasets (as per Fig. 1) were scanned for m5 (a) and Inr (b) core promoter elements, as described [46, 48, 61]. The number of genes transcribed by either element is shown. Every gene was trimmed from its putative TSS to the 100th nt in its ORF. Genes with high sequence similarity (> 90%) were clustered and represented by one single sequence. The sizes of the four non-redundant datasets are shown and putative transcripts in Datasets 1 and 4 were permuted 100 times. Genes in Dataset 1 were ranked based on the TE values and the top and bottom 200 genes were extracted. The general folding pattern of mRNAs in *T. vaginalis* was determined following a sliding window scheme and MFE calculation using the original and permutated sequences in the non-redundant Datasets 1 and 4. GC content across the length of transcripts was also analysed. Folding patterns of *T. vaginalis* genes with high and low TE values were compared, i.e. comparisons between Top vs. Bottom 200 genes from Dataset 1 and between genes in Dataset 2 vs. Dataset 3.

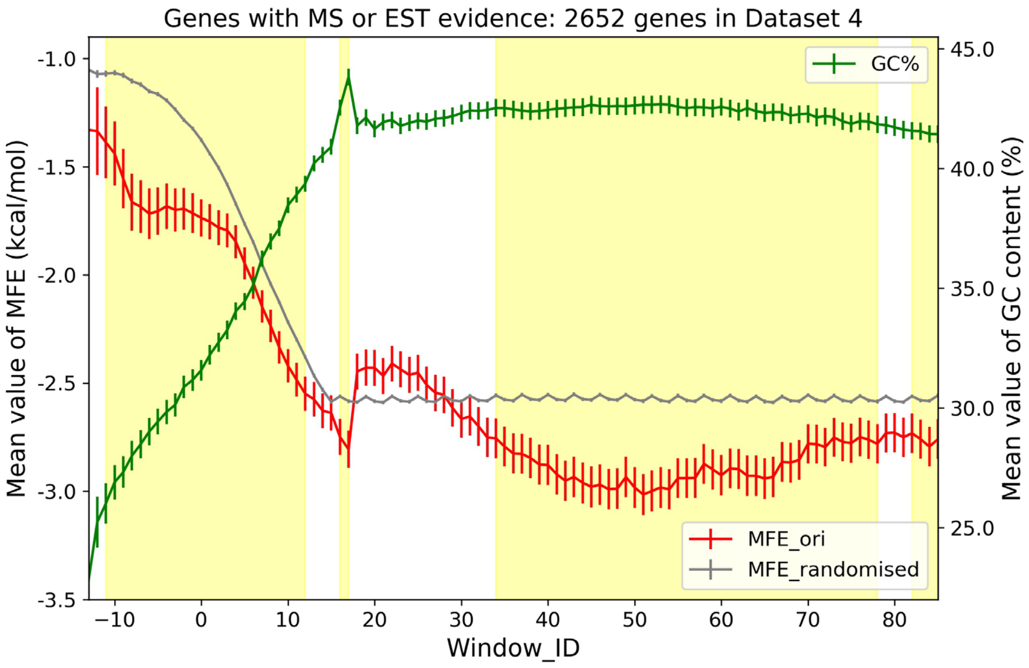

Fig. S3 Structural pattern and variation of GC content across the length of *T. vaginalis* mRNAs from Dataset 4. Following the exclusion of genes with unpredictable TSS and combination of genes with >90% sequence identity (Fig. S2), a total of 2,652 genes were included in this analysis. They were sliced into a series of 30 nt wide windows following a sliding window scheme. The index of a window was determined by the position of its central nucleotide relative to the start codon. The value on y axis is the mean MFE value (left) and GC percentage (right) of all sequences from the same window position and the error bar indicates the 95% confidence interval. Values of MFE and GC content for Dataset 1 and the MFE values of the permuted sequences were plotted (red, green and grey curves respectively). Independent t-tests were performed for the two MFE values (original vs. permutated) at the same window position and regions were shaded in yellow if adjusted p< 0.05 following a Bonferroni Correction (α=0.05).

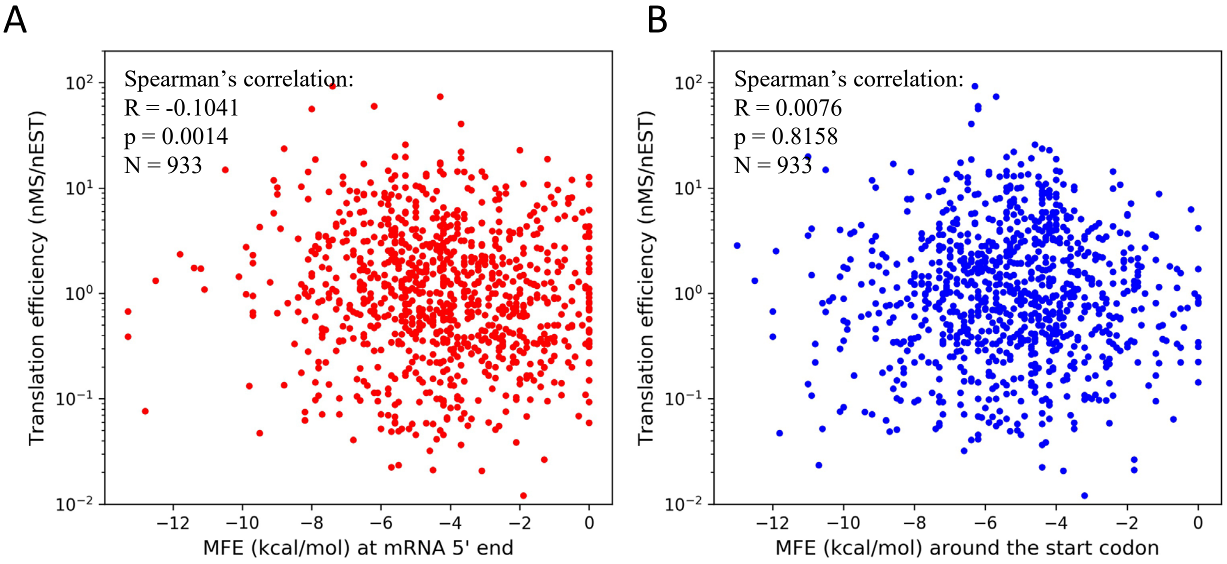

Fig. S4 Spearman's correlation analyses did not reveal a direct association between TE and MFE at 5′ end or surrounding the AUG start codon of *T. vaginalis* mRNAs from Dataset 1. Statistical analyses were carried out for the first 40 nt of the mRNAs (A) and for the region between -4 and +37 relative to the AUG start codon (B). The r, p values and sample size n are shown on the top left corners of the figures.

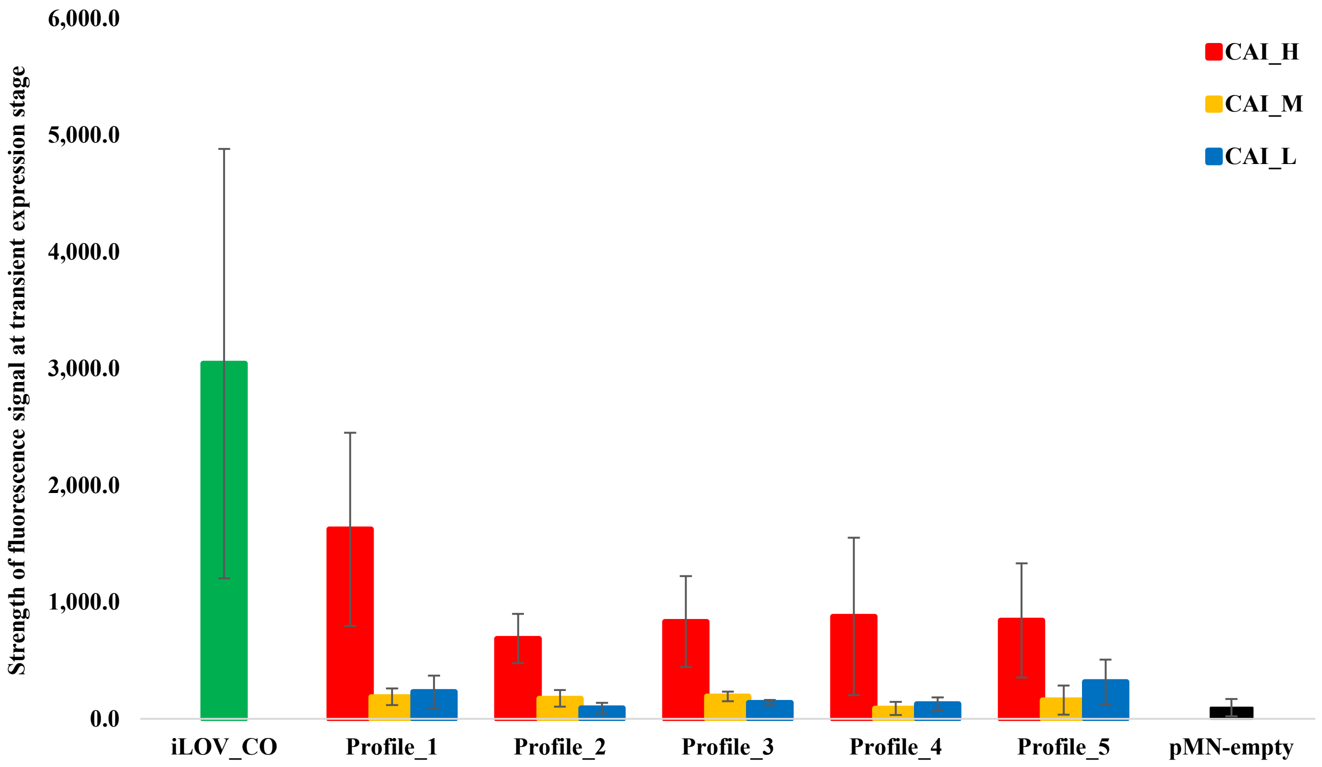

Fig. S5 Transient expression of the synonymous iLOV genes from three independent transfections. Percentage of dots (individual cells) located in quadrant Q3 were determined from each flow cytometry sample and the median fluorescence intensities of the dots in Q3 were calculated based on their values on FL1. Product of these two factors was calculated for each sample as a measurement of the strength of fluorescent signals released during transient expression. One-way ANOVA and Tukey's range test were conducted to determine the significance of differences between folding profiles and CAI levels. Results of the One-way ANOVA and the Tukey's range test are shown in Additional file: Description of Statistical Analyses and Table S1, respectively.

Table S1 Pairwise comparisons across transient expression illustrated on Fig. S5. A Tukey's range test was performed for all the strengths of fluorescent signals and a total of 75 adjusted p values were extracted from the test report. Each of the 15 synonymous iLOV genes was compared to the positive and negative controls (grey), i.e. iLOV_CO and pMN-empty respectively. Moreover, among the 15 synonymous iLOV genes, comparisons were made between each two CAI levels with the same 5′ folding profile (yellow) and between each two 5′ folding profiles with the same CAI level (green). Adjusted p values were colored in red if < 0.05.

Profile	CAI	Profile_1			Profile_2			Profile_3			Profile_4			Profile_5		
		CAI_L	CAI_M	CAI_H	CAI_L	CAI_M	CAI_H	CAI_L	CAI_M	CAI_H	CAI_L	CAI_M	CAI_H	CAI_L	CAI_M	CAI_H
1	L															
	M	0.9														
	H	0.2177	0.1807													
2	L	0.9														
	M		0.9		0.9											
	H			0.7785	0.9	0.9										
3	L	0.9			0.9											
	M		0.9			0.9		0.9								
	H			0.9			0.9	0.9	0.9							
4	L	0.9			0.9			0.9								
	M		0.9			0.9			0.9		0.9					
	H			0.9			0.9			0.9	0.9	0.9				
5	L	0.9			0.9			0.9			0.9					
	M		0.9			0.9			0.9			0.9		0.9		
	H			0.9			0.9			0.9			0.9	0.9	0.9	
iLOV_CO		0.001	0.001	0.1931	0.001	0.001	0.0011	0.001	0.001	0.0027	0.001	0.001	0.0035	0.001	0.001	0.0028
pMN-empty		0.9	0.9	0.1202	0.9	0.9	0.9	0.9	0.09	0.09	0.9	0.9	0.9	0.9	0.9	0.9

Table S2 Pairwise comparisons across stable expression illustrated on Fig. 5B. A Tukey's range test was performed for all the median stable fluorescence intensity values and a total of 75 adjusted p values were extracted from the test report. Each of the 15 synonymous iLOV genes was compared to the positive and negative controls (grey), i.e. iLOV_CO and pMN-empty respectively. Moreover, among the 15 synonymous iLOV genes, comparisons were made between each two CAI levels with the same 5′ folding profile (yellow) and between each two 5′ folding profiles with the same CAI level (green). Adjusted p values were colored in red if < 0.05.

Profile	CAI	Profile_1			Profile_2			Profile_3			Profile_4			Profile_5		
		CAI_L	CAI_M	CAI_H	CAI_L	CAI_M	CAI_H	CAI_L	CAI_M	CAI_H	CAI_L	CAI_M	CAI_H	CAI_L	CAI_M	CAI_H
1	L															
	M	0.001														
	H	0.001	0.1397													
2	L	0.9														
	M		0.0168		0.0248											
	H			0.0143	0.001	0.1585										
3	L	0.9			0.9											
	M		0.001			0.4079		0.7632								
	H			0.001			0.9	0.001	0.0103							
4	L	0.9			0.9			0.9								
	M		0.0883			0.9			0.1149		0.0139					
	H			0.9			0.0178			0.001	0.001	0.001				
5	L	0.9			0.9			0.9			0.8911					
	M		0.812			0.7076			0.0026			0.9		0.001		
	H			0.9			0.0034			0.001			0.9	0.001	0.001	
iLOV_CO		0.001	0.001	0.001	0.001	0.001	0.001	0.001	0.001	0.001	0.001	0.001	0.001	0.001	0.001	0.001
pMN-empty		0.001	0.001	0.001	0.001	0.001	0.001	0.001	0.001	0.001	0.001	0.001	0.001	0.0048	0.001	0.001

Description of Statistical Analyses

CAI values were calculated for the filtered sequences in the four datasets (Fig. S1). The 531 CAI values in Dataset 2 were compared to the 2,210 CAI values in Dataset 3 in an independent t-test using the Python function scipy.stats.ttest_ind (https://docs.scipy.org/doc/scipy/reference/generated/scipy.stats.ttest_ind.html). A Spearman's Correlation analysis was then performed for the 1,058 filtered genes in Dataset 1, between their CAI and TE values, using the Python function scipy.stats.spearmanr (https://docs.scipy.org/doc/scipy/reference/generated/scipy.stats.spearmanr.html).

The overall folding patterns of 713 *T. vaginalis* mRNAs and 71,300 permuted sequences were compared and are presented in Fig. 3A. To generate the 100 randomised sequences for each gene, we performed mononucleotide shuffling for the 5' UTR but triplet codon shuffling for ORF. We then calculated the MFE values at each window position (window size = 30 nt) for all sequences and obtained 713 values for the original transcripts and 71,300 values for the randomised transcripts. We conducted a t-test for the two datasets (713 original vs 71,300 randomised) at each window position, yielding 98 t-test results (window_-12 to window_85, including window_0). Bonferroni correction was carried out, using Python function statsmodels.stats.multitest.multipletests (https://www.statsmodels.org/stable/generated/statsmodels.stats.multitest.multipletests.html), with the p values from the 98 t-tests as input. Adjusted p values output from the package are presented. Yellow-shaded regions indicate significant regions ($p < 0.05$) after Bonferroni correction. Similarly, we performed the same statistical analyses for the 2,652 mRNAs in Dataset 4 and the 265,200 randomised sequences and plotted the result on Fig. S3.

We ranked the 933 *T. vaginalis* mRNAs in Dataset 1 (Fig. S2) based on their TE values and compared the folding patterns of the top 200 and bottom 200 sequences (Fig. 3B). We

calculated the MFE values for these 400 sequences at each window position (window size = 30nt) and compared the two datasets (Top 200 vs Bottom 200) using a t-test. We obtained 98 p values and applied Bonferroni correction (as above). Yellow-shaded regions indicate significant regions (adjusted $p < 0.05$) after Bonferroni correction.

We compared the folding patterns of 1,617 sequences in Dataset 3 and 361 sequences in Dataset 2 (Fig. S2) using a series of t-tests across the window positions, followed by a Bonferroni correction. Yellow-shaded regions indicate significant regions ($p < 0.05$) following Bonferroni correction.

We introduced each of the 17 plasmids to *T. vaginalis* in three independent transfection assays, yielding 51 lines. We collected transient and stable expression results for all 51 samples. Transient expression was observed both qualitatively and quantitatively. We determined the early expression of the green fluorescence by visually observing signal (dots) in quadrant Q3 (Fig. 5A). Transient expression was quantified by multiplying the percentage of dots located in Q3 with the median FL1 value of this population (i.e. dots in Q3) and shown in Fig. S5. To quantify stable expressions, we calculated the median FL1 value of the entire cell population following drug selection with G418.

To assess the expression of synonymous iLOV genes, we performed a one-way ANOVA on the transient and stable expression data. Analysis on transient data produced $F=5.6$, $> F_{crit}(16, 34)=1.95$ ($\alpha=0.05$), rejecting the null hypothesis that all the transfectants have equal transient expression levels. Likewise, analysis on stable data produced $F=82.3$, $> F_{crit}(16, 34)=1.95$ ($\alpha=0.05$), also rejecting the null hypothesis. These one-way ANOVA analyses were undertaken using Python function scipy.stats.f_oneway (https://docs.scipy.org/doc/scipy/reference/generated/scipy.stats.f_oneway.html), with the 17 groups of transient or stable data (each group has three values) as input.

We next conducted a post hoc test to make pairwise comparisons. A Tukey's range test was performed for transient and stable expression data separately. This test was undertaken using Python function statsmodels.stats.multicomp.pairwise_tukeyhsd (https://www.statsmodels.org/stable/generated/statsmodels.stats.multicomp.pairwise_tukeyhsd.html), with all 51 values and their group identifiers (e.g. iLOV_M2) provided. A report with 136 adjusted p values was generated. We present 75 adjusted p values in Table S1 and S2, and do not show the comparisons between sequences that differ in both folding and codon usage (e.g. iLOV_M1 vs iLOV_H2).
